# Syphilitic Sore of Urethra

**Published:** 1836

**Authors:** 


### Art. III.—Syphilitic Sore of the Urethra.

We had prepared notes of a case similar to that we are about to quote from that excellent periodical, the Medico-Chirurgical Review, believing that such are often mistaken for simple gonorrhœa, especially in the early stages, in the various cities and large towns on sea boards and water courses. The difficulties encountered in the first place by the writer corresponds so nearly with our own, that we have no hesitation in presenting it to our readers as one that may prevent them from making similar mistakes. Had not the symptoms changed, or rather the sore throat supervened, the reporter might, and doubtless would, have continued to treat it for months as he commenced, without performing a cure. He seems to have profited by the first case, for the next, though worse, was cured without any difficulty. We treated the one which fell under our notice for months as a simple gonorrhœa, without producing any sensible change, but we no sooner altered our plan than an amendment commenced, which resulted in an entire cure in the course of a few weeks.

W. W.

"The following are two examples of this rare affection. I say rare affection, because, though it is probable that venereal ulcerations occur now and then in the urethra, beyond the sphere of vision, the evidence of their existence is too slight to permit much confidence to be placed in it. In the following cases that evidence was sufficient to produce conviction in my mind, and it probably will not appear altogether inconclusive to my readers.

"The first case was that of an out-patient at the Lock Hospital. It is unfortunate that my notes of the case have been lost, but the following are the principal particulars.

"*Case* 1. A young man, a mechanic, applied to me on account of a discharge from the urethra, pain in micturition, and painful erections at night. Without making a minute examination of the penis, a precaution, however, which, should always be adopted whenever a venereal patient presents himself, I looked upon the case as one of gonorrhœa and treated it accordingly. But it did not yield to the remedies prescribed, and it appeared peculiarly obstinate. Nearly three months had elapsed from the first appearance of the disorder, and discharge still existed, with painful erections at night. The pain was referred to a spot in the urethra, distant about an inch from the orifice. Here I could feel distinct induration. But other symptoms had now occurred, which directed my attention more particularly to the induration I have mentioned. The patient complained of sore-throat, and superficial yellowish ulceration was visible upon the tonsils. An eruption had also appeared upon the skin. It consisted of slight depositions in the cutis of a brownish red color, desquamating freely. It was what may be called the syphilitic psoriasis. In order to ascertain as perfectly as possible the nature of the indurated spot in the urethra, which I suspected to be really a venereal ulcer, I separated the lips of the orifice as widely as possible, with a fine pair of dressing forceps, and was enabled to obtain a glimpse of the spot in question. It appeared to be distinctly ulcerated.

"Under these circumstances, I did not hesitate to prescribe a course of mercury. The patient was ordered to take the blue pill, and all local applications to the urethra were abandoned. Under this plan he rapidly improved, and ultimately he perfectly recovered. The discharge soon ceased, the induration subsided, the secondary symptoms disappeared, and in short, the patient appeared completely cured. Whether a subsequent return of the secondary symptoms took place, I am unable to determine, as I have not seen the patient since the period at which he was dismissed.

"*Case* 2. A gentleman consulted me about the middle of August of the present year, on account of a complaint under which he had been laboring for four or five months. He said that he had been treated for gonorrhœa.

"There was profuse purulent discharge from the urethra. On the right side of the orifice of the urethra was a distinct yellow ulceration, partly external, principally extending into the urethra. But this was not all. For two inches from the orifice the urethra felt hard, irregular and thickened, excessively painful upon pressure, and when pressed giving exit at the orifice to an increased quantity of discharge, slightly tinged with blood. On the breast and back was an eruption of ptyriasis, yet attended with rather more thickening of the cutis than is usual in that cutaneous affection. On the abdomen were several scattered spots, exactly resembling the syphilitic psoriasis. I told the gentleman I was confident he had ulceration of the throat, with such a combination of the symptoms. He said that he did not believe he had any thing the matter there. I examined the throat and found each tonsil extensively ulcerated. The ulceration was of a yellowish color, superficial, without much surrounding redness.

"The history of the case was indistinct. The patient had been living uninterruptedly with a female friend, but he had also had connexion at intervals with other women. It appeared that he had been subject for some time to ulcerations on the glans. He had had what was considered the gonorrhœa for four or five months, or more. The eruption on the skin had existed for two or three months also, but the spots of psoriasis had been of late occurrence. As he was not aware of the presence of ulceration in the throat, he was ignorant, of course, of the date of its occurrence.

"Under these circumstances, I could not hesitate to believe that the patient labored under syphilitic ulceration in the urethra. The eruption on the skin, the ulceration in the throat, the irregularity and tenderness of the urethra continuous with evident ulceration at the orifice, and finally the inutility of remedies directed for some time against the supposed gonorrhœa, appeared to form a chain of evidence strongly in favor of the syphilitic nature of the malady.

"In a paper in a former number of this Journal, I directed attention to a very troublesome complication or consequence of gonorrhæa, chronic inflammation of the corpus spongiosum urethræ. In that affection the corpus spongiosum may be felt indurated, is tender to the touch, keeps up the discharge from the urethra, and sometimes gives rise to abscess in the cellular tissue of the penis. But in chronic inflammation of the corpus spongiosum, there are never, so far as I have seen, secondary affections of the throat or skin, nor is it attended with ulceration at the orifice of the urethra.

"I prescribed a course of mercury for this gentleman—the application of mercurial ointment to the inferior surface of the penis—injections of dilute black-wash into the urethra—and the use of the tepid bath. At the date of this report (Sept. 1,) but a short time has elapsed since this plan of treatment was commenced. Yet the ulceration at the orifice of the urethra has healed—the induration of the corpus spongiosum has diminished, the eruption is fading, and the ulceration of the throat has decreased. So far as they go, these results are favorable to the view I have taken of the case."

